# Multilocus ISSR Markers Reveal Two Major Genetic Groups in Spanish and South African Populations of the Grapevine Fungal Pathogen *Cadophora luteo-olivacea*

**DOI:** 10.1371/journal.pone.0110417

**Published:** 2014-10-13

**Authors:** David Gramaje, Maela León, Marcela Santana, Pedro W. Crous, Josep Armengol

**Affiliations:** 1 Instituto Agroforestal Mediterráneo, Universidad Politécnica de Valencia, Valencia, Spain; 2 CBS-KNAW Fungal Biodiversity Centre, Utrecht, The Netherlands; USDA ARS, United States of America

## Abstract

*Cadophora luteo-olivacea* is a lesser-known fungal trunk pathogen of grapevine which has been recently isolated from vines showing decline symptoms in grape growing regions worldwide. In this study, 80 *C. luteo-olivacea* isolates (65 from Spain and 15 from South Africa) were studied. Inter-simple-sequence repeat-polymerase chain reaction (ISSR-PCR) generated 55 polymorphic loci from four ISSR primers selected from an initial screen of 13 ISSR primers. The ISSR markers revealed 40 multilocus genotypes (MLGs) in the global population. Minimum spanning network analysis showed that the MLGs from South Africa clustered around the most frequent genotype, while the genotypes from Spain were distributed all across the network. Principal component analysis and dendrograms based on genetic distance and bootstrapping identified two highly differentiated genetic clusters in the Spanish and South African *C. luteo-olivacea* populations, with no intermediate genotypes between these clusters. Movement within the Spanish provinces may have occurred repeatedly given the frequent retrieval of the same genotype in distant locations. The results obtained in this study provide new insights into the population genetic structure of *C. luteo-olivacea* in Spain and highlights the need to produce healthy and quality planting material in grapevine nurseries to avoid the spread of this fungus throughout different grape growing regions.

## Introduction

Grapevines are one of the most widely grown fruit crops in the world with significant plantings in Europe, North and South America, South Africa and Australasia. Grapes are used in the production of wine, brandy, or non-fermented drinks and are eaten fresh or dried as raisins [1]. The history of European grape-growing was characterized by a troubled second half of the 19^th^ century, in which European grape growers were faced with the arrival of three major phytosanitary problems: first powdery mildew, then phylloxera and, finally, downy mildew.

Powdery mildew caused by the fungus *Erisyphe necator,* was introduced from North America to England in 1845, and onwards to France in 1847. The root-feeding aphid called phylloxera was found in western France about 1865 and rapidly spread throughout Europe. In 1878, resistant rootstocks were introduced to Europe from North America in an effort to control this pest, but the downy mildew pathogen (*Plasmopara viticola*) was inadvertently introduced into France, probably as oospores on the imported rootstocks, and spread widely throughout Europe [2].

These past epidemics illustrate the destructive effects of new or introduced pathogens and the diseases they cause on grapevine cultivation. Fungal trunk diseases, namely esca, eutypa and Botryosphaeria diebacks, as well as black-foot and Petri diseases, are currently threatening the viticulture industry worldwide causing significant economic losses due to reduced yields, increased crop management costs for cultural and chemical preventive measures, and shortened life span of the vines [3], thus being named as the “Phylloxera of the 21^th^ century” [4]. Among them, Petri disease has emerged as one of the most devastating diseases affecting young vines in newly planted vineyards [5]. The ascomycete fungus *Cadophora luteo-olivacea* was traditionally considered as a minor vascular pathogen associated with Petri disease of grapevine. This fungus has been found in many habitats, such as decaying wood [6] and water [7] in the Antarctica, saline and acidic soils in the Czech Republic [8] or associated with decay symptoms in vines and fruit-rotting of kiwi in Italy [9]. However, in recent years *C. luteo-olivacea* has been increasingly reported in grapevine nurseries and young vineyards worldwide, causing a major concern in both grape growers and nurseries [10]–[15].

Halleen et al. [11] isolated 15 strains of *C. luteo-olivacea*, previously identified as *Phialophora* sp., from the graft union of apparently healthy plants in commercial nurseries in South Africa. The pathogenicity of one of these isolates was further demonstrated under glasshouse and field conditions [12]. Rooney-Latham [14] indicated that *C. luteo-olivacea* was quite common in esca and Petri-disease affected grapevines and isolated 13 strains throughout Californian vineyards. Gramaje et al. [10] recovered 58 *C. luteo-olivacea* isolates from grapevines showing black vascular streaking and decline symptoms characteristic of Petri disease, and from environmental samples collected at different growth stages of grapevines in Spanish nurseries. The pathogenicity of representative Spanish isolates was confirmed under field [16] and greenhouse conditions [10]. In Uruguay, Navarrete et al. [13] evaluated the pathogenicity of one strain of *C. luteo-olivacea* obtained from nursery planting material showing external and internal symptoms of Petri disease, but results were inconclusive. More recently, Úrbez-Torres et al. [15] isolated six strains of *C. luteo-olivacea* associated with young vine decline in British Columbia (Canada), and reproduced symptoms of dark vascular streaking on inoculated grapevine wood under greenhouse conditions.

Despite the clear implication of *C. luteo-olivacea* in early stages of grapevine wood disease, the biology, epidemiology and management of this fungus affecting grapevines remain poorly understood. An understanding of its mode of reproduction and the source of inoculum responsible for *C. luteo-olivacea* dispersal is, however, essential for the efficient management of Petri disease. To date, no sexual fruiting bodies of *C. luteo-olivacea* have ever been found in vineyards. However, sexual reproduction may be transient in vineyards or may occur on currently unidentified alternative host plants. In Spain, this species was isolated from asymptomatic xylem tissues of weeds collected in commercial vineyards [17], and it was also detected in natural soils from grapevine nurseries by using bait plants [18]. Gramaje et al. [19] investigated in vitro the sensitivity of *C. luteo-olivacea* to hot-water treatments and found that conidial germination of this fungus was inhibited at temperatures above 51°C, while treatments of up to 54°C for 60 min were necessary to inhibit mycelial growth.

Population genetics as applied to plant pathogens hold enormous promise for understanding certain evolutionary forces controlling pathogen populations (e.g., selection, migration, and recombination). Population genetic inference also provides insights into whether populations are sexual or clonal, differentiated or admixed, and whether phenotypes are linked to genotypes [20]. All of this knowledge is important for the development of improved integrated pest management strategies and successful resistance breeding programs. Control of endogenous pathogens such as *C. luteo-olivacea* in grapevines is problematic. In vineyards, management strategies recommended for prevention and disease management mainly involve the prevention and/or correction of predisposing stress situations [21]. Nursery vines have been identified as a significant source of Petri disease in vineyards around the world. There are many opportunities for infection by fungal trunk pathogens during the propagation process: wounds made in the tissue at every stage of production, hydration tanks or callusing rooms. Therefore, an integrated management program that includes HWT, chemical, biological, or other control measures has been suggested to be the most interesting procedure to reduce infections by fungal trunk pathogens during the nursery stages [5].

Multilocus molecular marker technology has been showed to be highly suited to assess the genetic structure of plant-pathogenic fungi [22]–[24]. In contrast to other markers such as virulence and fungicide resistance, molecular markers are presumed to be selectively neutral and therefore may be used to study evolutionary processes [25]. One approach, inter-simple-sequence repeat (ISSR)-PCR, is a discriminatory, genome-wide DNA fingerprinting method that involves amplification of a DNA segment flanked by two identical microsatellite repeats but oriented in opposite directions [26]. These simple-sequence repeats (SSRs) or microsatellites are hypervariable and are randomly distributed throughout the genome; the number of these repeating motifs will vary according to the individual. The main advantages of ISSR-PCR are that they do not require the knowledge of genome sequences, detection of high levels of polymorphism, low cost, simple operation, high stability and reproducibility, and rapid turnover [27]. Disadvantages include the fact that these are dominant markers precluding the possibility of detecting heterozygosity, lack of knowledge of allelic bands, interpreting alleles that are identical in state as being identical by descent, potential problems of contaminated template DNA or can have reproducibility problems [28].

To date, the only study of the biology and genetic structure of *C. luteo-olivacea* populations from grapevines was performed by Gramaje et al. [10], who obtained identical sequence profile among 58 Spanish *C. luteo-olivacea* isolates by amplification of the highly conserved ITS region, the partial β-tubulin (BT) or elongation-factor 1α (EF) genes, suggesting that the population of this species was represented by a single clonal lineage. The Spanish and South African *C. luteo-olivacea* isolates represent the unique collection of this fungus available for its use in population genetic studies. Therefore, the objectives of the present study were (i) to determine the genetic structure of *C. luteo-olivacea* in Spain at a regional scale by using multilocus ISSR markers, (ii) to compare the genetic structure of *C. luteo-olivacea* isolates from Spain and South Africa and (iii) to assess the virulence of representative *C. luteo-olivacea* isolates from the genetic clusters obtained in this study.

## Materials and Methods

### Ethics statement

No specific permits were required for the described field studies. Location of grapevine nurseries and vineyard sites were facilitated by regional Plant Health services in Spain and by ARC Infruitec-Nietvoorbij in South Africa. Permission to sample the vineyards and nurseries were granted by the landowner and nurserymen, who are collaborating with the Universidad Politécnica de Valencia (Valencia, Spain). The sites are not protected in any way. The areas studied do not involve any species endangered or protected in Spain and South Africa.

### Isolates

**Figure 1:**
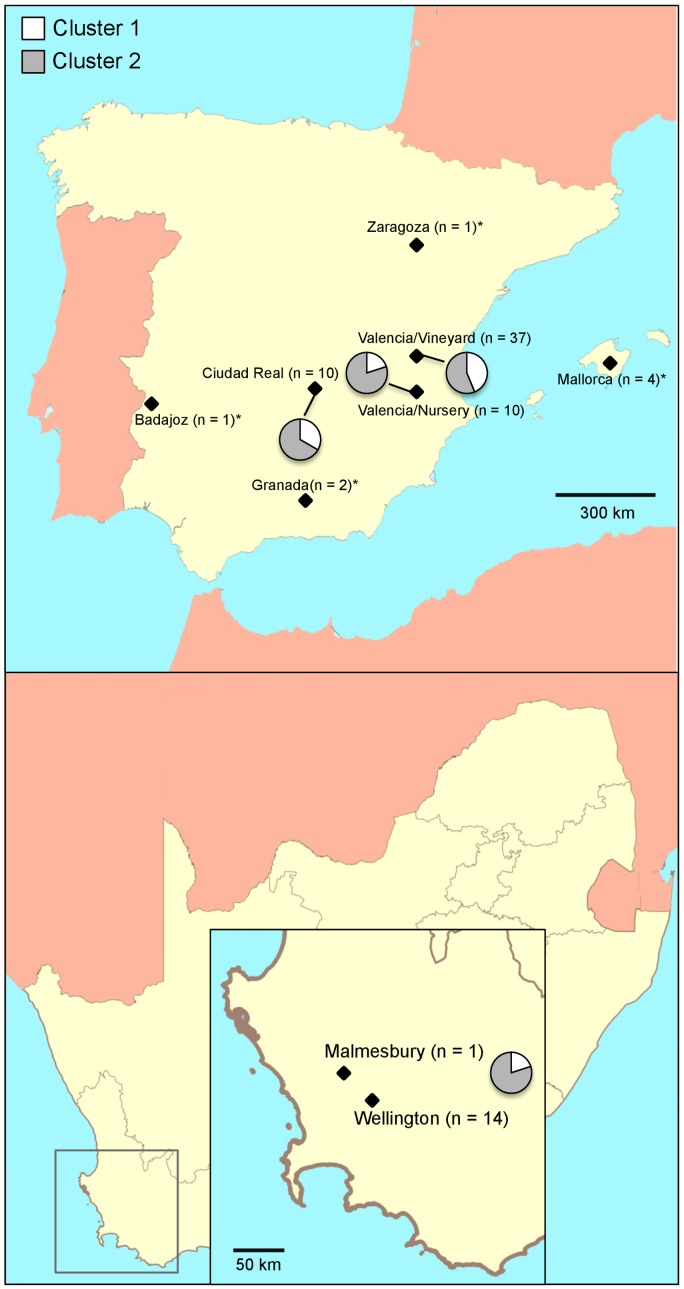
Geographic distribution of the Spanish and South African *Cadophora luteo-olivacea* isolates analyzed with four ISSR primers. The results of the PCA and dendrogram analyses of the Spanish and South African multi-locus genotypes (MLGs) are reported on the map: in white, MLGs assigned to cluster 1, in gray, MLGs assigned to cluster 2. The number of isolates for each population is indicated in brackets. Spanish populations with less than five individuals were not considered for all the analyses, and are indicated by an asterisk.

In this study 80 *C. luteo-olivacea* isolates were genotyped. The collection consisted of 65 isolates from Spain and 15 isolates from South Africa (Figure 1, Table 1). Spanish populations were sampled from rootstock wood of young plants showing black vascular streaking and decline symptoms characteristic of Petri disease (n = 55), and from nursery samples and planting material at different stages of the propagation process (n = 10). Vineyard isolates were collected between 2007 and 2010 from six provinces in Spain (Badajoz, Ciudad Real, Granada, Mallorca, Valencia and Zaragoza) (Figure 1, Table 1). Additionally, 10 isolates were collected in 2008 from four nurseries in Aielo de Malferit (located between Valencia and Alicante provinces) and treated as a single population. This is one of the main nursery areas in Spain and distributes planting material to all Spanish grapevine regions. Fifty-eight Spanish isolates were included in a previous study by Gramaje et al. [10]. The South African population was sampled in 1999–2000 from apparently healthy rooted cuttings in grapevine nurseries in Malmesbury (n = 1) and Wellington (n = 14) in the Western Cape Province (Figure 1, Table 1). These 15 isolates were identified previously as *Phialophora* sp. and reported by Halleen et al. [11].

**Table 1:** *Cadophora luteo-olivacea* isolates obtained from grapevine in Spain and South Africa included in this study^a^.

	Origin			
Country, province	Vineyard	Nursery	Number of isolates	Year
Spain^b^				
Badajoz	1	…	1	2007
Ciudad Real	10	…	10	2007–2010
Granada	2	…	2	2009
Mallorca	4	…	4	2008
Valencia	37	10	47	2008–2010
Zaragoza	1	…	1	2008
Subtotal	55	10	65	
South Africa	…	15	15	1999–2000
Total	55	25	80	

a Isolates from Spain were collected by D. Gramaje. South African isolates were collected by F. Halleen from grapevine nurseries in Malmesbury (1 isolate) and Wellington (14 isolates).

b Fifty-eight Spanish isolates were included in a previous study by Gramaje et al. [10].

*Cadophora luteo-olivacea* isolates were collected from rootstock sections in Spain and rooted cuttings in South Africa as previously described by Gramaje et al. [10] and Halleen et al. [11], respectively. Samples from Spanish nurseries were taken at three stages of the propagation process: samples from pre-grafting hydration tanks, washings from scissors and washings from grafting tools. Water samples were collected and filtered as described by Aroca et al. [29]. The isolates were single-spored by serial dilution [30] and identified according to their morphological characters as well as the sequence analyses of the ITS region, BT and EF genes prior to use [10]. They were then stored in a 15% glycerol solution at −80°C in 1.5 mL cryovials.

### DNA isolation and quantification

Freeze-dried fungal tissue was ground to a fine powder under liquid nitrogen using a mortar and pestle. Total DNA was extracted using the E.Z.N.A. Plant Miniprep Kit (Omega Biotek, Doraville, GA) following the manufacturer’s instructions. DNA was quantified using a spectrophotometer (ND-2000, NanoDrop Technologies, Wilmington, DE) and adjusted to a final concentration of genomic DNA at 15 ng µl^–1^ for PCR amplification.

### ISSR profiling

Fifteen *C. luteo-olivacea* isolates were used in a preliminary screen to determine those ISSR primers that enabled generation of polymorphic, reproducible markers that could be used to generate polymorphic DNA fingerprints for all *C. luteo-olivacea* isolates. This screen tested 13 ISSR primers (Table 2), which were previously reported to produce polymorphic and reproducible DNA fingerprints from other fungi and plant species. Each PCR reaction contained 1X PCR buffer, 2.5 mM MgCl_2_, dNTPs at 100 µM each, primers at 0.4 µM each, 0.5 U DNA Taq polymerase and 0.5–5 ng template DNA. The PCR reaction mix was adjusted to a final volume of 25 µL with water (Chromasolv Plus, Sigma-Aldrich). PCR amplifications were performed in a Peltier Thermal Cycler-200. Conditions included an initial step of 5 min at 95°C, followed by 34 cycles of denaturation at 95°C for 1 min, annealing at primer-specific temperature (Table 2) for 1 min, and elongation at 72°C for 2 min. A final extension was performed at 72°C for 10 min. PCR products were visualized in 1.5% agarose gels (agarose D-1 Low EEO, Conda). The Gene Ruler 100-bp DNA ladder plus was used as a molecular weight marker (Fermentas Inc., Hanover, MD, USA). Analysis was replicated at least three times for the subset of isolates, with independent DNA extractions, PCR, and sizing of fragments to confirm reproducibility of results. Those primers that generated reproducible, clearly discernible, polymorphic bands in repeated experiments were selected to subsequently amplify genomic DNA from all isolates.

**Table 2:** Band characteristics of the four inter-simple-sequence repeat (ISSR) primers that were selected and ultimately used in repeated experiments^a^.

ISSR primer	Ta(°C)^b^	N° of amplified bands	Polymorphism (%)	Primer description
(GAC)_5_	46.0	20	80	Anchorless tri-nucleotide
(ACTG)_4_	48.0	12	91.7	Anchorless tetra-nucleotide
(GACA)_4_	46.0	22	86.4	Anchorless tetra-nucleotide
DDB(CCA)_5_	61.0	13	84.6	Tri-nucleotide, 5′ anchor present

a ISSR primers that were unable to generate polymorphic and reproducible markers: (GAA)_6_, (GGAT)_4_, (GATA)_4_, DBDA(CA)_7_, YHY(GT)_5_G, HBH(AG)_7_A, BDB(ACA)_5_, DHB(CGA)_5_ and DBH(TCG)_5_.

b Annealing temperature.

Amplification profiles of the 80 *C. luteo-olivacea* isolates generated by the four selected ISSR primers were compared and the DNA fragments were scored computationally using the GelAnalyzer 2010a software (http://www.gelanalyzer.com). Only bands (size: 100 to 2,500 bases) that could be scored consistently for all samples were used, with the assumption that each band with different molecular weight represented a distinct locus and amplicons sharing the same molecular weight were considered to be the same allele at a specific locus. The absence of amplicons was considered as an alternate allele.

### Data analysis

Amplified DNA fragments were transformed into a binary character matrix (1 =  presence, 0 =  absence). A multilocus genotype (MLG) was constructed for each isolate by combining data for single ISSR fingerprints by using the procedure available in the package POPPR [31] for R version 3.0.3 (The R Foundation for Statistical Computing) [32]. Isolates with the same MLG were considered clones, and some analyses were conducted for the global and clone-corrected data set.

To assess the possible evolutionary relationships among MLGs, minimum spanning networks were constructed, first from genotypes of different provinces or nurseries in Spain (nursery is considered as a single population separate from the vineyard subpopulations by provinces) and also including the genotypes from South Africa. Relative dissimilarity distances were calculated according to the index of association [33]. It returns a distance reflecting a ratio of the number of observed differences by the number of possible differences. The number of possible differences is the number of loci multiplied by ploidy. The R package POPPR [31] was used to calculate the dissimilarity distance matrices and to generate minimum spanning networks from the matrices.

We tested for the existence of divergent genetic pools of *C. luteo-olivacea* in Spain using a genetic multivariate analysis [34] and a dendrogram including bootstrap support for clades to detect genetically differentiated groups. These methods avoid the clustering of individuals on a priori knowledge such as geographical locations that may mix divergent genetic lineages introduced in the same area and may hinder the detection of admixture events among these lineages. First, we used a principal component analysis (PCA) to investigate the genetic structure of the *C. luteo-olivacea* population in Spain, and the global population in Spain and South Africa. PCA is independent of any genetic hypotheses and it is suitable for the analysis of partially clonal species. PCA analysis was performed using the R package ADEGENET [35]. For this analysis, only single copies of the different genotypes were used to give identical weight to MLGs. Populations with less than five individuals (Badajoz, Granada, Mallorca and Zaragoza) were not considered for the analyses. UPGMA dendrograms were also inferred from the distance matrices and visualized using Molecular Evolutionary Genetic Analysis (MEGA) software, version 6 for Windows [36]. Bootstrapping was performed with the R package Pvclust with 10,000 bootstrap resamplings [37]. Populations with less than five individuals (Badajoz, Granada, Mallorca and Zaragoza) were not considered for all the analyses.

The genetic richness (R) and the evenness index adapted from the Simpson index of genotypic diversity (ED*) were calculated for each population using the R package POPPR [31]. Genotypic diversity (D) was calculated using D = n/(n–1)(1–Σpi^2^) as implemented in MULTILOCUS [38], where *pi* is the frequency of the *i*th genotype and n is the number of individuals sampled. D represents the probability that two individuals sampled at random with replacement have the same genotype, and is similar to the diversity measure of Pielou [39]. Rarefaction curves representing species richness were calculated to determine if the sampling intensity was adequate to detect the majority of *C. luteo-olivacea* strains present in each population. Because sample size varied among populations we employed rarefaction to explore the effect of sample size on observed species richness [40]. The function ‘rarecurve’ from the R package vegan [41] was used to generate rarefaction curves.

To study the contribution of possible sexual reproduction to the genotypic diversity observed for each population, the probability that a genotype was obtained by chance throughout a sexual event (P_gen_) was calculated using GENCLONE [42], as well as the probability that an isolate shared the same MLG as another in the sampled population (P_sex_), assuming that the two isolates derive from sexual reproduction [43]. Linkage disequilibrium as an indication of random mating was calculated and tested for significance with 1,000 randomizations using the R package POPPR [31]; measures of gametic disequilibrium tested were the index of association (*I*_A_), and a standardized alternative of the *I*_A_ (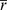
_d_) [38]. The null hypothesis for this test is that there is random association among alleles at different loci and *I*_A_ = 0; the null hypothesis for random mating is rejected where *I*_A_>0. These tests were not performed on samples sizes <10 due to lack of statistical power [44].

Population genetic structure was analyzed by conducting an analysis of molecular variance (AMOVA) [45] on clone-corrected data using ARLEQUIN version 3.5 [46]. The fixation index (F_ST_) was also calculated using ARLEQUIN. Significance of F_ST_ was tested using 1,000 permutations. Due to low sample sizes, populations from Badajoz, Granada, Mallorca and Zaragoza were excluded from the analysis.

### Virulence assays

Representative isolates of *C. luteo-olivacea* were randomly selected from each of the two genetic clusters (6 isolates representing 4 MLGs from cluster 1 and 8 isolates representing 5 MLGs from cluster 2) inferred by the Bayesian clustering method and the genetic multivariate analysis. Virulence assays were conducted on 1-year-old grapevine cuttings of 110 Richter (R) rootstock. In total 168 dormant cuttings were cut into uniform lengths containing four to five buds, and then hot-water treated at 53°C for 30 min to eliminate the possible incidence of fungal trunk pathogens [47]. In order to enhance callusing and rooting, dormant cuttings were buried into sterilized peat moss in plastic boxes, and placed in a callusing room at 25°C and 100% humidity for 4 weeks. After callusing and rooting, cuttings were wounded between the two upper internodes with a 5 mm cork borer. A 5 mm mycelium agar plug from a 2-weeks-old culture was placed in the wound. Wounds were wrapped with Parafilm. Six cuttings per fungal isolate were inoculated with 5 mm uncolonized PDA plugs from two different plates as negative controls. Inoculated cuttings were planted immediately in individual pots, placed in a greenhouse at 25°C and watered every 3 d or as needed. Plants were arranged in a completely randomized design. The experiment was repeated.

Cuttings were collected after 4 months and inspected for lesion development. Extent of vascular discoloration was measured upward and downward from the inoculation point. Additionally, shoot dry weight was evaluated for sprouts formed above the inoculation point. Small pieces (0.5 to 1 cm) of necrotic tissue from the edge of each lesion were cut and plated onto malt extract agar (MEA) (Oxoid Ltd., Basingstoke, Hants, England) supplemented with 0.5 g L^−1^ streptomycin sulphate (MEAS) (Sigma-Aldrich, St. Louis, MO, USA) in an attempt to recover the inoculated fungi and confirm Koch’s postulates. Fungi were identified as previously described.

Analyses of variance (ANOVA) were conducted to analyze lesion length and shoot dry weight data. Homogeneity of variance was tested using Levene’s test. Residuals were visually inspected for each treatment, and when necessary a log_10_ transformation was used to improve homogeneity of variance. Treatment means among individuals and genetic clusters were compared using Student’s least significant difference (LSD) test at *P*<0.05. In all cases the Statistix 10 software (Analytical Software, FL, USA) was used.

## Results

### ISSR markers

In total, 55 polymorphic loci were generated from ISSR-PCR using the four ISSR primers selected from a screen of 13 ISSR primers (Table 2). The highest number of polymorphic bands (PBs) was produced for primer (GACA)_4_ (an anchorless tetra-nucleotide, 19 PBs), followed by primer (GAC)_5_ (an anchorless tri-nucleotide, 16 PBs), primer (ACTG)_4_ (an anchorless tetra-nucleotide) and primer DDB(CCA)_5_ (a 5′-anchored tri-nucleotide), both with 11 PBs. The mean percentage of polymorphic loci was 85.65%.

### Genotypic diversity

In general, Spanish subpopulations showed high genotypic diversity. The four ISSR primers generated a total of 31 MLGs among the 65 Spanish *C. luteo-olivacea* isolates (Table 3). The most frequent MLG was MLG31 (18.4%), which was present in subpopulations from nursery, Mallorca and Valencia, followed by MLG14 (12.3%) which was present in nursery subpopulation and Ciudad Real and Valencia provinces, and MLG29 (12.3%), containing only isolates from Valencia province. MLG13 (10.8%) was also frequent and was detected in Ciudad Real and Valencia provinces. Of the 31 MLGs, two were observed twice (MLG3 in Mallorca region and MLG26 in Valencia province) and the remaining MLGs were observed once (80.6% of the MLGs). When including the 15 isolates from South Africa in the analysis, the four ISSR primers generated a total of 40 MLGs. Of the 10 MLGs observed in the South African subpopulation, the most frequent MLG was MLG13 (40.0%). The remaining 9 MLGs were observed once.

**Table 3:** Genetic diversity of the Spanish and South African populations of *Cadophora luteo-olivacea* contrasting regional vineyard populations (Ciudad Real and Valencia), the nursery population from Valencia region and populations between countries^a^.

Parameters^b^	Total	Spain	Ciudad Real	Valencia	Nursery	South Africa
Sample Size (N)	80	65	10	37	10	15
Genotypes (G)	40	31	8	17	5	10
Genotypic richness (R)	0.49	0.47	0.78	0.44	0.44	0.64
Evenness (ED*)	0.55	0.60	0.93	0.65	0.62	0.63
Diversity (D)	0.93**	0.92**	0.95**	0.89**	0.67**	0.86**

a The nonredundant indices of genotypic diversity recommended by Arnaud-Haond et al. [43] were calculated for each population on the total data set.

b R, genotypic richness, R = (G–1)/(N–1) where G is the number of multilocus genotypes discriminated; ED*, evenness index adapted from Simpson diversity; D, genotypic diversity. ** = *P*<0.01.

**Figure 2:**
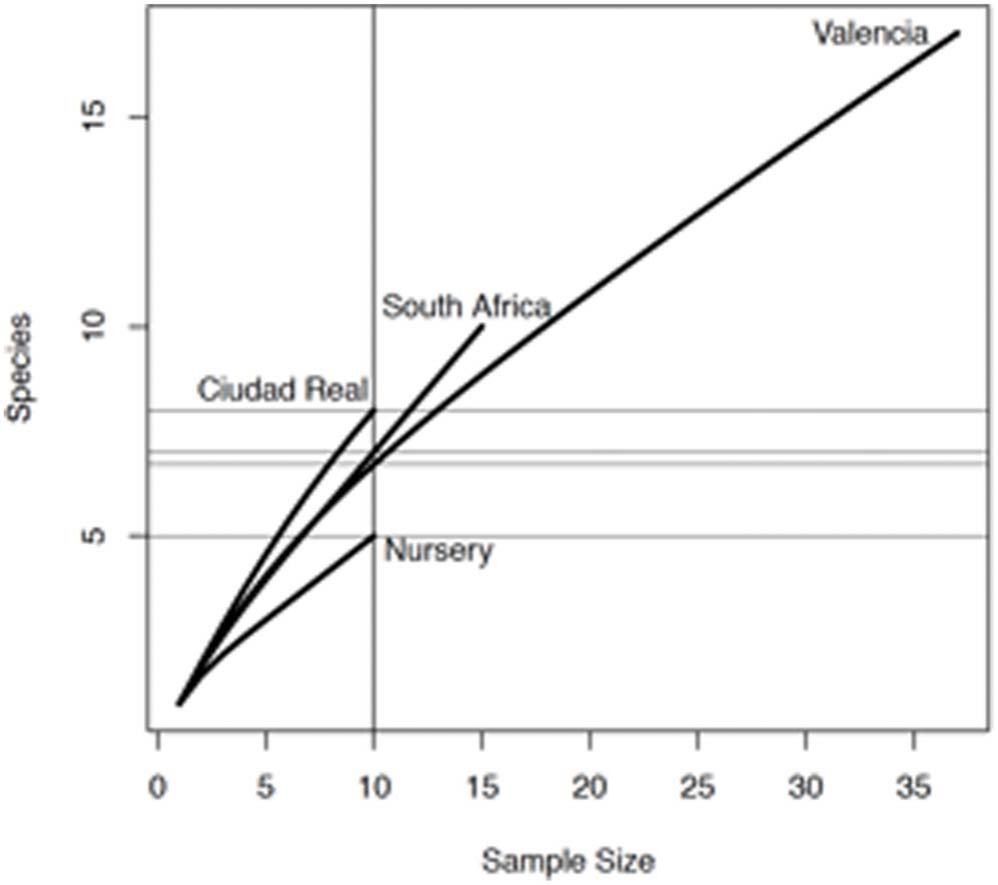
Rarefaction curves for sampling *Cadophora luteo-olivacea* isolates in Ciudad Real and Valencia provinces, in nursery subpopulation and in South Africa.

Overall, genetic richness (R) varied across subpopulations defined by different provinces in Spain and South Africa, from very high in Ciudad Real or South Africa (0.64 to 0.78), to high in Valencia or the nursery subpopulation (0.44) (Table 3). Evenness (ED*) values were higher in the Ciudad Real subpopulation (0.93) compared with the regional populations in Spain and South Africa (0.62–0.65). Diversity (D) values varied from very high (0.95) in the Ciudad Real subpopulation, where nearly all individuals were a distinct genotype, and in Valencia and South Africa (0.86 to 0.89); to high for the nursery subpopulation, where five genotypes were detected within the 10 isolates sampled. According to the rarefaction curves (Figure 2), Valencia province had a higher number of samples and observed taxa relative to the other subpopulations. A sample size of 10 (the sample size for Ciudad Real province and nursery subpopulation) was common to all populations. Comparison of rarefaction curves showed significant differences in diversity among these populations at this common sample size (Figure 2) with Ciudad Real province being more diverse than the others, and with South Africa and Valencia province having comparable amounts of diversity as well as being more diverse than nursery subpopulation.

### Relationship among genotypes

**Figure 3:**
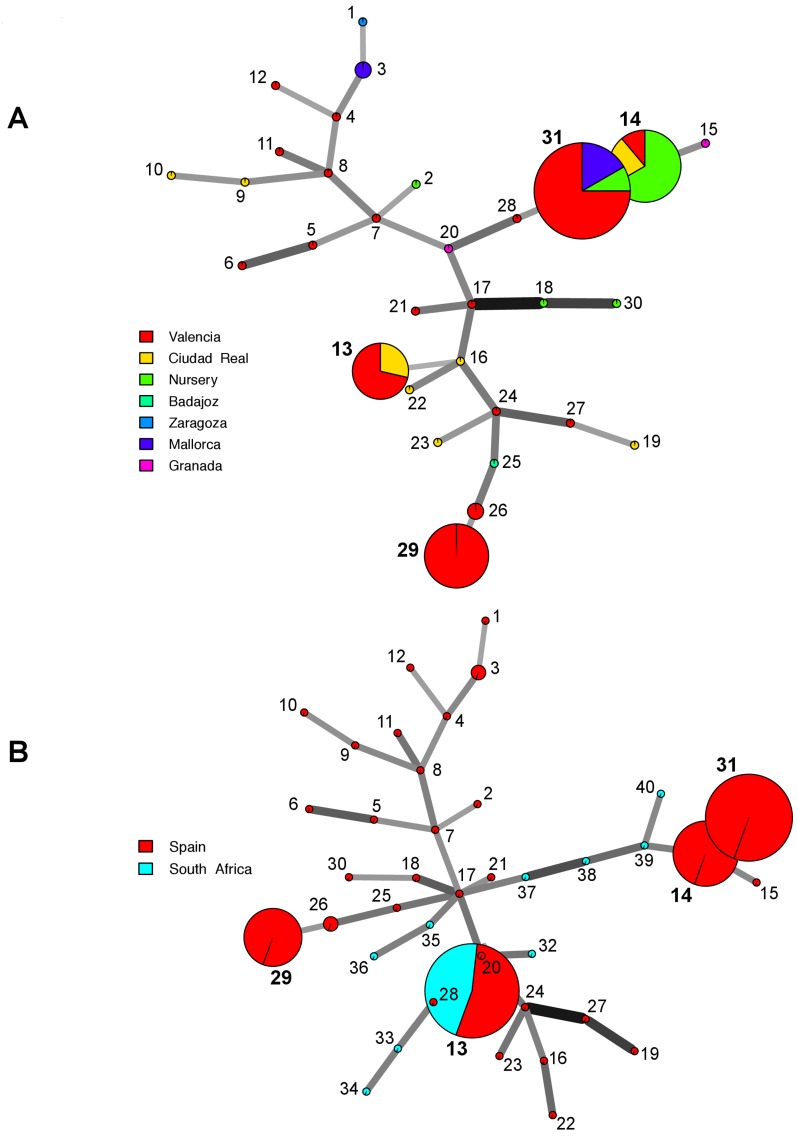
Minimum Spanning Network showing the relationship among the individual multi-locus genotypes (MLGs) observed in A, the Spanish population of *Cadophora luteo-olivacea* and B, the populations from Spain and South Africa. Each node represents a different MLG. Node sizes and colors correspond to the number of individuals and population membership, respectively. Edge thickness and color are proportional to absolute genetic distance. Edge lengths are arbitrary. The four most common MLGs (13, 14, 29 and 31) are outlined in the graphs.

The minimum spanning network for the Spanish population distinguished the most frequent MLGs in Spain, MLG13, MLG14, MLG29 and MLG31 (Figure 3A). The global MLG network showed that the MLGs from South Africa clustered around the most frequent genotype (MLG13), while the genotypes from Spain were distributed all across the network (Figure 3B).

### Population stratification

**Figure 4:**
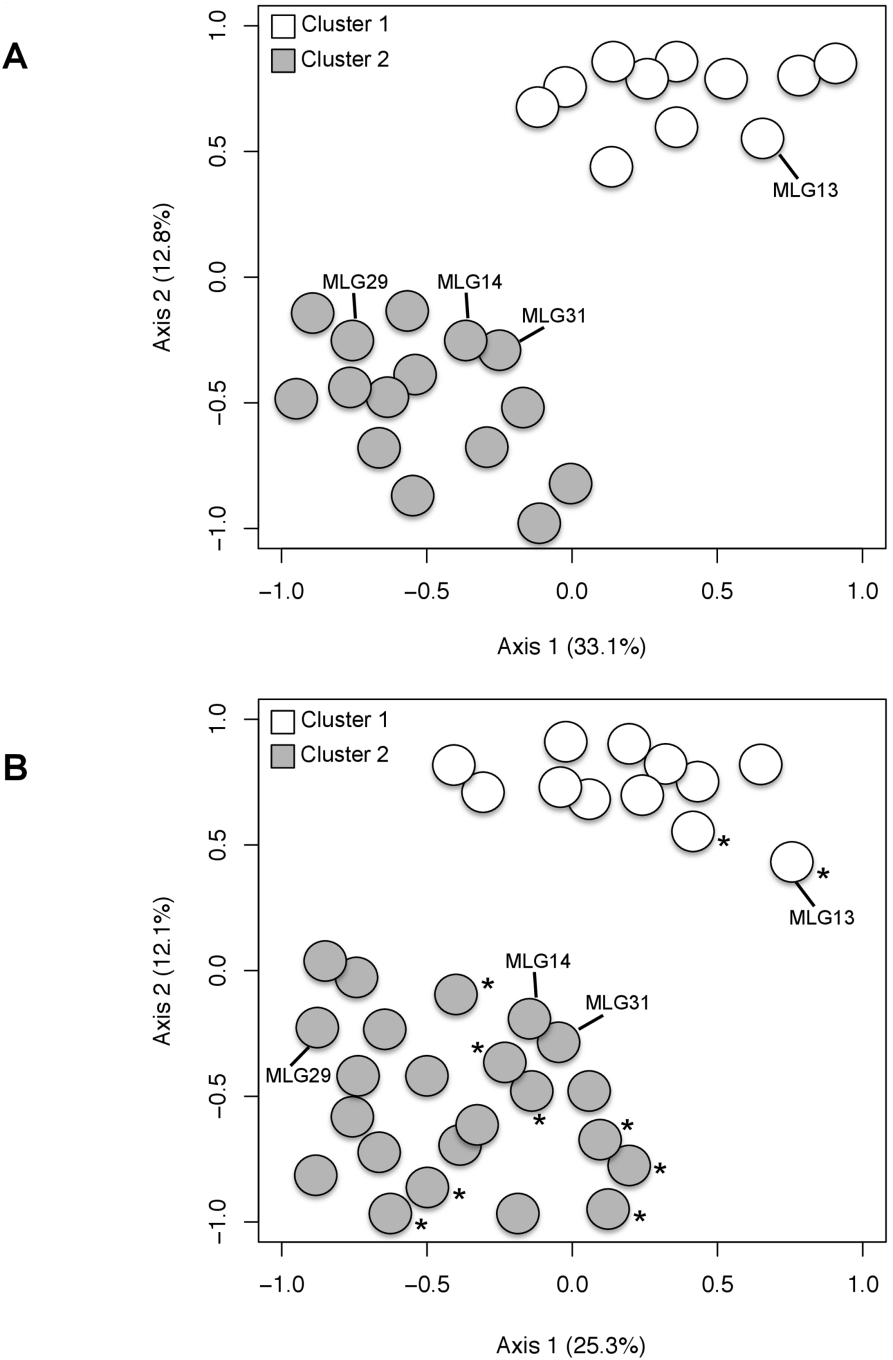
Coordinates of A, the 26 different *Cadophora luteo-olivacea* genotypes sampled in Spain and B, the 35 genotypes sampled in Spain and South Africa on the two main axes of the PCA. The four most common MLGs (13, 14, 29 and 31) are outlined in the graphs. Asterisk (*) indicates the observed MLGs in South Africa. MLG13 was observed in Spain and South Africa. Spanish populations with less than five individuals were not considered for the analysis.

**Figure 5:**
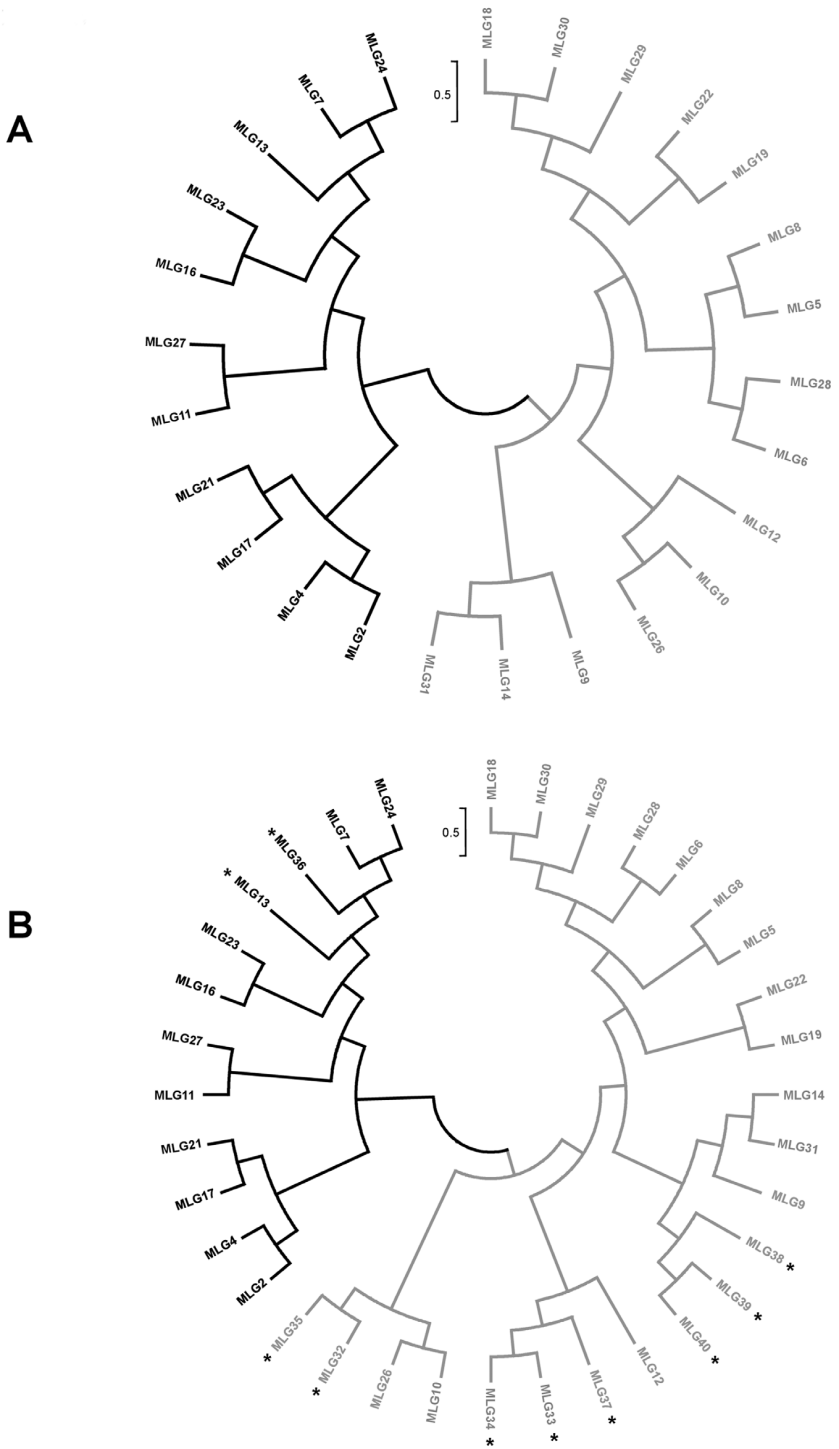
UPGMA dendrogram of genetic distance among A, 26 observed multilocus genotypes (MLGs) in Spain and B, 26 Spanish MLGs and 10 observed MLGs in South Africa. Clusters are colored according to PCA analyses results: in black, MLGs assigned to cluster 1, in gray, MLGs assigned to cluster 2. Support values greater than 70% using 1,000 bootstrap samples are shown. Asterisk (*) indicates the observed MLGs in South Africa. MLG13 was observed in Spain and South Africa. Spanish populations with less than five individuals were not considered for the analysis.

Principal component analysis revealed two clusters of MLGs (Figure 4a). Axis 1 and 2 of the PCA accounted for 33.1% and 12.8% of total genetic variability. Regarding the analysis of the dataset from Spain and South Africa, 12 MLGs were grouped in one cluster, containing two MLGs from South Africa, namely MLG13 and MLG36 (Figure 4b). A second cluster was composed of 23 MLGs, and included eight MLGs from South Africa. Principal component analysis also revealed two clusters of MLGs (Figure 4b). Axis 1 and 2 of the PCA accounted for 25.3% and 12.1% of total genetic variability. Data from the genetic distance matrices were analyzed using an UPGMA algorithm; only the optimal UPGMA trees are presented (Figure 5). The two genetic clusters were separated by large genetic distances, with robust bootstrap (BS) support for separation among clusters (BS>70%). In both analyses, the clusters discriminated using the dendrogram analysis were in agreement with the clusters inferred using multivariate approach (Figure 4).

The AMOVA results based on Spanish populations (Table 4), showed 6.3% of the variation among vineyard populations considered together regardless of geographical origin and nursery subpopulations, 5.5% among populations within vineyard and nursery environments, and 88.1% within populations. The overall F_ST_ was 0.062 and the permutation *P* value was 0.03. The AMOVA results based on populations defined by isolates’ country of origin (Table 4) showed 13.3% of the variation among populations and 86.7% within populations. The overall F_ST_ was 0.135 and the permutation *P* value was <0.00001. The two clusters identified in multivariate and dendrogram clustering analyses in the Spanish *C. luteo-olivacea* population were also tested for significance and apportionment of variation with hierarchical AMOVA; wherein 23.8% of the variation was apportioned among the two clusters and 76.2% within clusters (Table 4). The overall F_ST_ was 0.238 and the permutation *P* value was <0.00001.

**Table 4:** Analysis of molecular variance (AMOVA) testing for genetic differentiation between regional populations of *Cadophora luteo-olivacea* in Spain, populations defined by country of origin and genetic clusters obtained in Spain.

Source of variation	Variation (%)	F_ST_	*P* a
Regional populations within Spain			
Among vineyard populations regardless thegeographical origin and nursery population	6.3	…	…
Among populations within vineyard and nursery	5.5	…	…
Within populations	88.1	0.062	0.03
Global populations by country of origin			
Among populations	13.3	…	…
Within populations	86.7	0.135	<0.00001
Grouped by genetic clusters in Spain			
Among clusters	23.8	…	…
Within clusters	76.2	0.238	<0.00001

a F_ST_, fixation index; significance was determined by 1,000 permutation.

### Selfing and clonality in populations

The linkage disequilibrium tests (*I*_A_ and 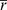
_d_) on all individuals and the clone-corrected data rejected the null hypothesis for recombination (*P*<0.01) (Table S1). The probabilities that the most frequent genotypes detected in Spain and South Africa evolve by chance through sexual reproduction (P_gen_) and that two isolates share these genotypes in the Spanish and South African populations (P_sex_) were very low (Table S2).

### Virulence assays

**Figure 6:**
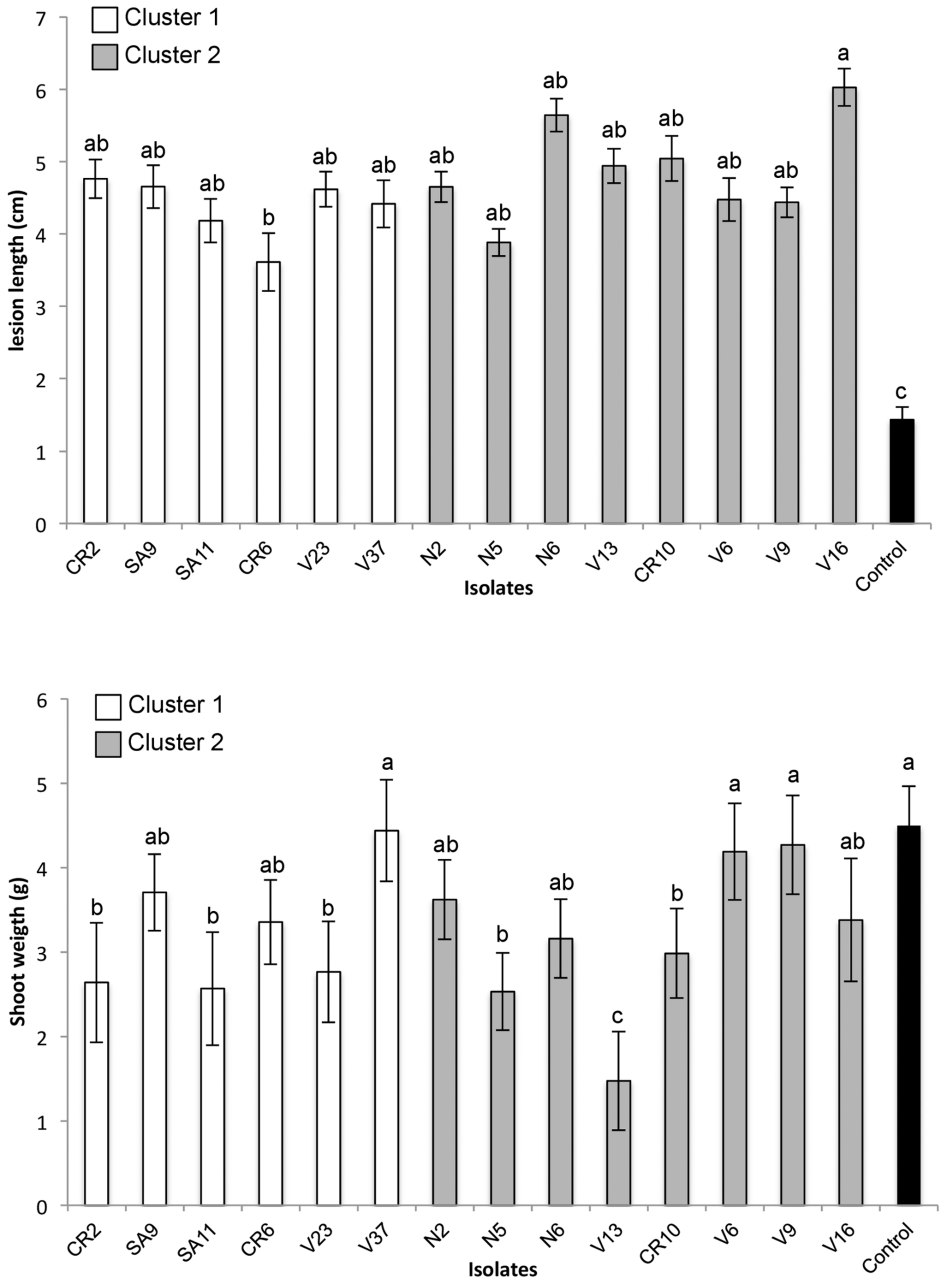
Virulence tests of *Cadophora luteo-olivacea* isolates from the two genetic clusters generated from Bayesian and PCA analyses. Cluster 1 was represented by 4 MLGs (MLG 13: isolates CR2, SA11 and SA9; MLG16: isolate CR6, MLG4: isolate V23; MLG27: isolate V37). Cluster 2 was represented by 5 MLGs (MLG 14: isolates N2 and N5; MLG31: isolates N6 and V13; MLG19: isolate CR10; MLG12: isolate V6; MLG29: isolates V9 and V16). A, Mean lesion length caused by 14 isolates in 110 R rootstock 4 months after inoculation. B, Mean shoot weight caused by 14 isolates in 110 R rootstock 4 months after inoculation. Mean lesion length and shoot weight are based on twelve replicates per isolate. Means followed by different letters differ significantly (P<0.05) according to Student’s least significant difference test. Bars represent standard error of the mean.

Data of the two virulence assays were combined because of the lack of significant differences between the two tests and among the studied variables (*P*>0.05). All isolates of *C. luteo-olivacea* used in this study were pathogenic to grapevine cuttings of 110 R rootstock (Figure 5). Symptoms developed 4 months after inoculation consisted of leaves with interveinal chlorosis and necrosis, and necrotic xylem lesions. The statistical analysis indicated significant (*P*<0.05) differences in virulence among treatments. Values of lesion length ranged between 3.6 and 6.0 cm, and the values of shoot dry weight from 1.5 to 4.6 g. All the *C. luteo-olivacea* isolates caused lesions in the xylem of grapevine rootstock that were significantly longer than in the control. The average shoot dry weight of the isolates CR2, SA11, V23, N5, V13 and CR10 was significantly different compared to the control (Figure 6). Treatment means among isolates belonging to the two genetic clusters showed no significant differences when evaluating the lesion length (*P* = 0.2628; Cluster 1: 4.38±0.35; Cluster 2: 4.77±0.38) and the shoot weight (*P* = 0.9894; Cluster 1: 3.19±0.28; Cluster 2: 3.19±0.23).

## Discussion

This study is the first to employ genetic markers to analyze the population structure of a collection of *Cadophora luteo-olivacea* isolates obtained from vineyards and grapevine nurseries from different provinces in Spain and South Africa. Clustering analysis (PCA and dendrograms based on genetic distance and bootstrapping) identified two highly differentiated genetic clusters in the Spanish *C. luteo-olivacea* population with no intermediate genotypes among these clusters, one including a common MLG, namely MLG13, and the second including the other three dominant genotypes, namely MLG14, MLG29 and MLG31. Movement of MLGs within the Spanish provinces may have occurred repeatedly, given the low level of geographic genetic structure and the frequent retrieval of the same genotype in distant locations. Highly similar, but not identical, clonal genotypes are unlikely to have arisen independently in sexual reproduction and can then be inferred to represent the same clonal lineage [48], [49].

The two genetic groups displayed no specific geographic distribution, thus suggesting clonal divergence and admixture between the Spanish *C. luteo-olivacea* genotypes. In fact, AMOVA analyses showed that most genetic diversity (88.1%) was found within populations and only 5.5–6.3% among populations. The two defined clusters would then correspond to an established asexual lineage intermingled with individuals derived from another asexual lineage that were recently admixed. Providing that sexual reproduction is a rare and episodic event in *C. luteo-olivacea* [10], such admixture processes are expected to maintain their signature in populations for several generations. There are several other documented examples of asexual plant pathogens consisting of genetically differentiated clusters, such as *Phytophthora ramorum* [50], *Puccinia striiformis* [51], *Fusarium circinatum* [52], or the main fungal species associated with Petri disease and esca of grapevine, *Phaeomoniella chlamydospora* [53].

In our study, the hypothesis of a clonal population structure is supported by several population genetic criteria pointed out by Tibayrenc et al. [54]: (i) Overrepresented, widespread identical genotypes. Several MLGs were observed with widespread geographical distribution across Spanish vineyards. The most frequent genotype, MLG31, was found in nursery subpopulation and Valencia province. Other dominant genotypes in Spain were found in nurseries and in Ciudad Real and Valencia provinces (MLG14), and in Valencia and Ciudad Real provinces (MLG13). This can be explained by the existence of an efficient long distance dispersal means through infected plant material. Contributions of inoculum from plant material in grapevine nurseries are well known, as *C. luteo-olivacea* has been detected in different phases of the propagation process in South Africa and Spain [10]–[12]. The high proportion of the most frequent genotypes and the P_sex_ values associated with these genotypes also provide evidence for clonal reproduction in the Spanish populations studied. (ii) Linkage disequilibrium tests. The null hypothesis of random mating was rejected for all subpopulations in Spain and South Africa, even when populations were clone corrected. However, caution is required in the interpretation of these findings, since it is difficult to demonstrate the presence of linkage disequilibrium where sample sizes are small. (iii) Finally, sexual structures of *C. luteo-olivacea* have never been observed under natural conditions and all attempts to induce sexual morph in culture were unsuccessful [10].

The high levels of genotypic diversity found in this study could be explained by the higher evolutionary rate of change within microsatellite regions compared with other regions. The hypervariable nature of ISSR markers enables detection of a higher level of polymorphism compared with other molecular markers [28], [55]. High percentages of polymorphic loci were obtained using ISSR markers in previous studies [22]–[24]. Under conditions of strict clonal reproduction, high levels of gene flow together with a large effective population size would be required to account for such genotype diversity. Similar results were obtained by Comont et al. [53], when studying the population genetic structure of the asexual fungal species *Phaeomoniella chlamydospora*. Given the high haplotype diversity together with linkage disequilibrium, these researchers suggested that *P. chlamydospora* could be an asexual species possibly undergoing rare recombination or partial recombination due to parasexuality. Continued genotyping of *C. luteo-olivacea* from vineyards and nurseries will be necessary to confirm the results of genotype diversity, to track the movement and diversification of the lineages and to identify new dominant genotypes or newly introduced lineages.

The South African genotypes also grouped into the two genetic clusters obtained in the analysis of the Spanish *C. luteo-olivacea* population using a multivariate approach. Additional sampling of *C. luteo-olivacea* isolates from South Africa and other countries will be necessary to confirm that the global population of this species consists in two different clonal lineages. MLG13, which was one of the most common MLG in Spain, was found relatively frequently in South Africa. It is worth noting that this MLG was found 8 years apart: namely, in 1999 in Wellington (South Africa) and again, widely distributed in Valencia and Ciudad Real provinces, in 2007–2008. This demonstrates that the fungus can be dispersed on infected plant material for long distance introductions.

There is currently no information on populations of *C. luteo-olivacea* in other grape growing regions to help evaluate how different they may be from the Spanish population. *Cadophora luteo-olivacea* has been recently associated with Petri disease, one of the most important diseases of young vines [5]. This new occurrence in nurseries and vineyards, together with the fact that it is very difficult to obtain the number of isolates required for population studies, could explain the lack of studies of the genetic variability of this pathogen. Sampling for this fungus requires the destruction of the plant, because it grows in and around the xylem vessels of the grapevine trunk. In our study, isolate collection accounted for more than 4 years of vineyard and nursery work in Spain. The low recovery rate of *C. luteo-olivacea* is in accordance with published literature [10]–[15]. Population genetic analyses of *C. luteo-olivacea* isolates from other grape growing regions of the world will contribute significantly to better understand the reproductive system of this species, which might be crucial for planning successful control strategies.

The results obtained here provide new insights into the population genetic structure of *C. luteo-olivacea* in Spain. Over the past few years this pathogen has been shown to be involved with young grapevine decline in many grape growing regions worldwide. This study revealed the existence of a genetic admixture, with the presence of two divergent and well-differentiated genetic groups corresponding to two sympatric clonal lineages, each composed of closely related clonal variants. The epidemiological importance of these genetic clusters in relation to the different levels of virulence was investigated. All isolates were able to induce typical Petri disease symptoms in xylem vessels of 110 R rootstock. However, no association could be found between virulence phenotype and genetic cluster. Our findings highlight the need to produce healthy and quality planting material in grapevine nurseries to avoid the dispersal of trunk disease pathogens throughout different grape growing regions.

## Supporting Information

Table S1
**Estimates of linkage disequilibrium (** ***I*** **_A_ and 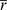
_d_) within Spanish subpopulations, countries and genetic clusters in Spain.**
(DOC)

Table S2 **Probability of occurrence or a second encounter of multilocus ISSR genotypes observed more than once in the regional populations studied in Spain and South Africa.**
(DOC)
