# Supplementary material for: Multilocus ISSR Markers Reveal Two Major Genetic Groups in Spanish and South African Populations of the Grapevine Fungal Pathogen Cadophora luteo-olivacea
Source: PLoS One. 2014 Oct 13;9(10):e110417. doi: 10.1371/journal.pone.0110417 (PMC4195744; doi:10.1371/journal.pone.0110417)
Supplement: Table S1 — Estimates of linkage disequilibrium ( I A and d) within Spanish subpopulations, countries and genetic clusters in Spain. (DOC) [file pone.0110417.s001.doc]

**Table S1**. Estimates of linkage disequilibrium (*I*_A_ and $\overline{r}$_d_) within Spanish subpopulations, countries and genetic clusters in Spain^a^

|  |  | All isolates | | |  |  | Clone corrected | | |
| --- | --- | --- | --- | --- | --- | --- | --- | --- | --- |
| Populations | *N* | *I*_A_ | $\overline{r}$_d_ | *P* value |  | *N* | *I*_A_ | $\overline{r}$_d_ | *P* value |
| Ciudad Real | 10 | 1.417 | 0.035 | <0.01 |  | 8 | nc | nc | nc |
| Valencia | 37 | 3.941 | 0.085 | <0.01 |  | 17 | 1.258 | 0.026 | <0.01 |
| Nursery | 10 | 7.958 | 0.298 | <0.01 |  | 5 | nc | nc | nc |
| Spain | 65 | 2.582 | 0.049 | <0.01 |  | 31 | 1.092 | 0.021 | <0.01 |
| South Africa | 15 | 5.784 | 0.148 | <0.01 |  | 10 | 2.461 | 0.064 | <0.01 |
| Total | 80 | 2.442 | 0.043 | <0.01 |  | 40 | 1.086 | 0.019 | <0.01 |
| Cluster 1 | 24 | 1.112 | 0.056 | <0.01 |  | 12 | 0.658 | 0.017 | <0.01 |
| Cluster 2 | 48 | 1.002 | 0.061 | <0.01 |  | 23 | 0.616 | 0.018 | <0.01 |

^a^ Tests were performed on all and clone-corrected data but excluded sample sizes of <10 individuals. Values that differ significantly from 0 indicate a departure from linkage disequilibrium; nc, cannot be calculated.
