# Supplementary material for: Multilocus ISSR Markers Reveal Two Major Genetic Groups in Spanish and South African Populations of the Grapevine Fungal Pathogen Cadophora luteo-olivacea
Source: PLoS One. 2014 Oct 13;9(10):e110417. doi: 10.1371/journal.pone.0110417 (PMC4195744; doi:10.1371/journal.pone.0110417)
Supplement: Table S2 — Probability of occurrence or a second encounter of multilocus ISSR genotypes observed more than once in the regional populations studied in Spain and South Africa. (DOC) [file pone.0110417.s002.doc]

**Table S2.** Probability of occurrence or a second encounter of multilocus ISSR genotypes observed more than once in the regional populations studied in Spain and South Africa

| Population | MLG^a^ | N_obs_^b^ | P_gen_^c^ | P_sex_^d^ |
| --- | --- | --- | --- | --- |
| Valencia | 13 | 5 | 1.19 × 10^-7^ | 4.39 × 10^-6^ |
|  | 26 | 2 | 4.63 × 10^-8^ | 1.71 × 10^-6^ |
|  | 29 | 8 | 4.59 × 10^-9^ | 1.70 × 10^-7^ |
|  | 31 | 9 | 9.21 × 10^-14^ | 3.41 × 10^-12^ |
| Nursery | 14 | 6 | 5.88 × 10^-9^ | 0.00076 |
| Ciudad Real | 13 | 2 | 3.31 × 10^-8^ | 3.29 × 10^-7^ |
|  | 14 | 2 | 4.86 × 10^-11^ | 3.90 × 10^-10^ |
| Mallorca | 3 | 2 | 7.63 × 10^-6^ | 3.05 × 10^-5^ |
|  | 31 | 2 | 7.63 × 10^-6^ | 3.05 × 10^-5^ |
| South Africa | 13 | 6 | 6.95 × 10^-11^ | 1.04 × 10^-9^ |

^a^ MLG, multilocus genotype.

^b^ Number of times when the genotype was observed in the population.

^c^ Probability of the MLG occurring in the population and is calculated as the product of the allele frequencies.

^d^ Probability of obtaining the observed number of identical genotypes in the population as a result of random mating.
